# Emotional abuse and neglect: time to focus on prevention and mental health consequences

**DOI:** 10.1192/bjp.2020.154

**Published:** 2020-11

**Authors:** Veena Kumari

**Affiliations:** Division of Psychology, Department of Life Sciences, Brunel University London; and Centre for Cognitive Neuroscience, College of Health, Medicine and Life Sciences, Brunel University London, UK

**Keywords:** Childhood experience, emotional abuse, emotional neglect, mental health problems, resilience

## Abstract

Emotional abuse and emotional neglect are among the most prevalent of childhood maltreatment types and associated with a range of poor mental health outcomes. We need to move beyond correlational research and shift our focus to sophisticated multimodal studies to fully understand the psychobiological mechanisms underlying these associations and to intervention studies.

Childhood maltreatment is commonly described as physical, sexual or emotional abuse and physical or emotional neglect by a parent, caregiver or other adult, with all kinds of abuse resulting from acts of commission, and neglect from acts of omission. There has been relatively less societal and research attention on emotional abuse and emotional neglect, compared with physical and sexual abuse and physical neglect. This might be explained, at least in part, by their less visible immediate impact (i.e. no physical injury or outward signs of abuse) and considerable regional and cultural variations in the definition of what constitutes emotional abuse or (emotional) neglect. Certain facets of emotional abuse, such as constant swearing, yelling, criticism or humiliation of a child, are easily noticeable, but others, such as unrealistic expectations or unreasonable demands on the child, or unfair treatment because of certain characteristics (e.g. physical disability, or appearance), are not always recognised. In some cases, these less apparent facets of emotional abuse may arise out of the childhood or lived experience of parents, caregivers, teachers and others, but nonetheless still cause (unintended) harm to the child. Emotional neglect, defined usually as a failure to attend to the child's emotional needs (e.g. never showing emotion while interacting with the child), can also be difficult to spot and quantify, as many parents or caregivers find it hard to provide a safe and loving environment for their children when facing relationship difficulties, mental health problems or addiction issues. In some settings, emotional abuse/neglect may also echo culturally accepted practices, for example the girl child neglect phenomenon seen in certain Asian communities, whereby girls receive less care and fewer resources than boys in the same family. Elimination of childhood maltreatment, if (at all) possible, is going to require efforts from many sections of society, including the government. Until these efforts completely succeed, psychiatry, psychology and neuroscience disciplines must continue to act to fully understand the psychobiological processes that explain mental health problems emerging in association with emotional abuse/neglect, alone or simultaneously with other kinds of maltreatment, and to develop and test suitable interventions to correct them.

## The scale of the problem

Despite difficulties in recognising and measuring emotional abuse, meta-analyses of the global prevalence of maltreatment convincingly reveal that childhood emotional abuse is self-reported by a much larger proportion of the adult population (about 36%) compared with physical (about 18%) or sexual abuse (8–18%), or physical neglect (about 16%).^1,2^ Interestingly, studies reliant on informants of abuse have documented a much lower prevalence of emotional abuse than those using self-reports. Childhood emotional neglect, which is likely to be underreported in some settings, is still reported by about 18% of the adult population.^2^ Children from any background can experience emotional abuse/neglect, although the prevalence rates may be higher in certain groups. For example, lesbian, gay, bisexual or transgender (LGBTQ+) youth may be more prone to experiencing emotional abuse, and possibly all types of abuse, because of societal ignorance or non-acceptance. Children from disadvantaged sectors of society, such as child workers or children displaced owing to war and other crises, may be subject to both inter- and intra-familial abuse and neglect as they spend time away from their families. At present, there are limited data on this topic from specific subgroups or low-resource countries.

## Unpacking mental health consequences

Children who suffer maltreatment of any kind are known to experience poorer physical and mental health as adults, regardless of culture and geographical variations. Many people who suffered emotional abuse as children show feelings of hopelessness, poor self-esteem, reduced sense of social support, poor satisfaction with life, neurobiological changes in stress response systems, and structural and functional brain deficits; they are also at a heightened risk of developing psychiatric disorders. Problems such as depression, anxiety, eating disorders, suicidal symptomatology, psychosis, personality disorder and substance misuse often emerge in childhood and last through adulthood to old age. Importantly, a growing body of literature from both high- and low-income countries indicates that emotional abuse might have the most wide-ranging negative mental health impact of all childhood maltreatment types.^3^ At present, there are few data addressing mental health consequences of emotional versus physical neglect. Nonetheless, both emotional abuse and emotional neglect seem to be a transdiagnostic risk factor for psychiatric disorders, especially anxiety and depression, perhaps mediated by dysfunctional (emotional) processing of self- and other-related information, accompanied with altered use or reduced availability of neural resources.

Further to simple association studies, there is now a pressing need for further research to fully examine the mental health consequences of emotional abuse and neglect at both ‘what’ and ‘how’ levels (i.e. what are the behavioural and brain changes following emotional abuse/neglect and how do they contribute to specific mental health outcomes?). A clear understanding of the psychobiological mechanisms that mediate between childhood emotional abuse and neglect and later vulnerability to specific mental disorders is critical for reducing such vulnerability and identifying targets for developing novel interventions. Although there have been some studies of neurophysiological correlates of childhood maltreatment (e.g. event-related brain potentials to facial expressions of anger or fear in maltreated versus non-maltreated children, or adults with and without a history of childhood maltreatment), they typically have not distinguished between different types of abuse and neglect. They have neither specifically focused on emotional abuse/neglect, which can be present with or without physical and sexual abuse and physical neglect, nor examined the observed neurophysiological changes in relation to the risk for particular disorders (e.g. depression versus psychosis). There is also a need for longitudinal studies examining the long-term impact of specific abuse and neglect, along with associated psychobiological changes, on prevalence of psychiatric disorders that usually emerge later in life. Lastly, future studies must proactively enquire about the protective factors that might promote resilience in the face of childhood emotional abuse/neglect.

## Reversing adverse mental health effects

With a paucity of studies empirically addressing the mechanisms underlying the association between emotional abuse/neglect and mental disorders, there are few clearly defined targets for reversing, or preventing the risks for, mental health problems in emotionally maltreated youth. However, it is already known that adults who suffered childhood maltreatment in general show a worse-than-usual response to standard pharmacological approaches to ameliorate their mental health problems, such as depressive symptoms, and respond relatively better to psychological interventions. This, taken together with evidence (e.g.^3^) of the extensive and undesirable mental health impact of emotional abuse and neglect, encourages the development and use of psychological interventions, especially those targeting aberrant emotional processes, to reverse or even prevent (if applied in time) adverse mental health outcomes for maltreated children.

Encouragingly, there are early indications that psychological interventions aimed at correcting aberrant attentional processes or interpretational biases may be applied to improve mental health outcomes in maltreated youth.^4^ However, much of the research in this area has been correlational. The research focus and funding priorities now need to be expanded to include intervention studies^5^ and facilitate studies that would yield valuable information for identifying specific treatment targets (e.g. certain information processing or memory biases, unhelpful coping styles) for developing novel interventions and refining existing ones.

## Reducing harm through prevention strategies

To minimise the short- and long-term harm associated with emotional abuse and neglect, child and adolescent psychiatrists, clinical psychologists and other professionals who routinely work with young people need to actively look for their signs and intervene to educate and safeguard where indicated. It is also important for these disciplines to interact with law makers and enforcement bodies to ensure that emotional abuse and (emotional) neglect, in the absence of visible signs, are appropriately acknowledged in governmental policies, and that no historically underserved populations (e.g. girls in certain societies) are ignored. Armed with empirical evidence, mental health professionals should also be encouraged and empowered to actively contribute to grass root campaigns to raise public awareness about the signs and consequences of these extremely harmful forms of childhood maltreatment.

## Conclusions

Given the high prevalence rates and well-documented harmful mental health consequences of emotional abuse and neglect across countries and cultures, it is essential that we not only learn to recognise their signs but focus our efforts on clearly understanding the underlying mechanisms and on developing suitable interventions to minimise and prevent the risk of associated poor mental health outcomes. At present, the research on possible interventions for reversing the mental health problems associated with this kind of childhood maltreatment is in its infancy but shows promise. In parallel, we must also work towards raising public awareness about the signs and the mental health impact of emotional abuse and neglect and ensure that they are appropriately acknowledged in global child protection laws and policies.

## Supplementary material

For supplementary material accompanying this paper visit https://doi.org/10.1192/bjp.2020.154. click here to view supplementary material

## References

[1] Stoltenborgh M, Bakermans-Kranenburg MJ, Alink LRA, van IJzendoorn MH. The prevalence of child maltreatment across the globe: review of a series of meta-analyses. Child Abuse Rev 2015; 24: 37–50.

[2] Stoltenborgh M, Bakermans-Kranenburg MJ, Van Ijzendoorn MH. The neglect of child neglect: a meta-analytic review of the prevalence of neglect. Soc Psychiatry Psychiatr Epidemiol 2013; 48: 345–55. 22797133 10.1007/s00127-012-0549-y PMC3568479

[3] Pandey R, Gupta S, Upadhyay A, Gupta RP, Shukla M, Mishra RC, Childhood maltreatment and its mental health consequences among Indian adolescents with a history of child work. Aust N Z J Psychiatry 2020; 54: 496–508. 32156147 10.1177/0004867420909524 PMC7227131

[4] Lau JYF, Sharma NP, Bennett E, Dhakal S, Vaswani A, Pandey R, Acceptability of a brief training programme targeting attention and interpretation biases for threat in youth with a history of maltreatment. Behav Cogn Psychother 2020; 48: 370–5. 31708006 10.1017/S1352465819000663

[5] Berthelot N, Lemieux R, Maziade M. Shortfall of intervention research over correlational research in childhood maltreatment: an impasse to be overcome. JAMA Pediatr 2019; 173: 1009–10. 10.1001/jamapediatrics.2019.1684 31524929

